# Post-Stroke Functional Changes: In-Depth Analysis of Clinical Tests and Motor-Cognitive Dual-Tasking Using Wearable Sensors

**DOI:** 10.3390/bioengineering11040349

**Published:** 2024-04-02

**Authors:** Masoud Abdollahi, Ehsan Rashedi, Pranav Madhav Kuber, Sonia Jahangiri, Behnam Kazempour, Mary Dombovy, Nasibeh Azadeh-Fard

**Affiliations:** 1 Department of Industrial and Systems Engineering, Rochester Institute of Technology, Rochester, NY 14623, USA; ma8489@rit.edu (M.A.); pmk2015@rit.edu (P.M.K.); sj1374@rit.edu (S.J.); bk9656@g.rit.edu (B.K.); nafeie@rit.edu (N.A.-F.); 2 Department of Rehabilitation and Neurology, Unity Hospital, Rochester, NY 14626, USA; mary.dombovy@rochesterregional.org

**Keywords:** stroke, neurological disorders, movement analysis, kinematics, rehabilitation

## Abstract

Clinical tests like Timed Up and Go (TUG) facilitate the assessment of post-stroke mobility, but they lack detailed measures. In this study, 21 stroke survivors and 20 control participants underwent TUG, sit-to-stand (STS), and the 10 Meter Walk Test (10MWT). Tests incorporated single tasks (STs) and motor-cognitive dual-task (DTs) involving reverse counting from 200 in decrements of 10. Eight wearable motion sensors were placed on feet, shanks, thighs, sacrum, and sternum to record kinematic data. These data were analyzed to investigate the effects of stroke and DT conditions on the extracted features across segmented portions of the tests. The findings showed that stroke survivors (SS) took 23% longer for total TUG (*p* < 0.001), with 31% longer turn time (*p* = 0.035). TUG time increased by 20% (*p* < 0.001) from STs to DTs. In DTs, turning time increased by 31% (*p* = 0.005). Specifically, SS showed 20% lower trunk angular velocity in sit-to-stand (*p* = 0.003), 21% longer 10-Meter Walk time (*p* = 0.010), and 18% slower gait speed (*p* = 0.012). As expected, turning was especially challenging and worsened with divided attention. The outcomes of our study demonstrate the benefits of instrumented clinical tests and DTs in effectively identifying motor deficits post-stroke across sitting, standing, walking, and turning activities, thereby indicating that quantitative motion analysis can optimize rehabilitation procedures.

## 1. Introduction

Stroke remains a challenge for healthcare professionals in the 21st century, ranking among the foremost causes of disability in the adult population with ~795,000 new cases each year [1,2,3]. More importantly, two-thirds of surviving patients, amounting to ~7 million individuals in the United States alone, struggle with compromised mobility. Even after completing a rehabilitation program, only 40% of stroke survivors (SS) are able to completely reclaim their lost functional capabilities [4]. These losses include diminished sensory functions, as well as a decline in both motor and cognitive capabilities. Notably, post-stroke hemiparesis, a condition characterized by weakness on one side of the body, is common. This condition significantly impedes the ability to perform daily tasks, even the most fundamental tasks, such as getting in and out of bed independently. Such activities are collectively referred to as ‘Activities of Daily Living’ (ADLs). The impact of post-stroke changes in body movement, including aspects such as increased risk of fall, asymmetry, and reduced speed while performing ADLs, has been investigated in the literature [5,6,7]. These alterations remain a topic of interest in the stroke research community because they can increase the risk of injury to SS who are elderly (~70 years on average), making even the most trivial injuries fatal. Beyond physical movement, stroke also affects an individual’s cognitive performance, affecting sensory function and coordination, as noted by Dennis et al. [8]. The interconnectedness of neuro-muscular systems often compromises the ability to perform tasks that demand both motor and cognitive functions [9]. Effective monitoring and assessment of these functions can not only help in maintaining proper care but can also serve as a vital tool for ensuring effective rehabilitation of such patients.

Screening of deficiencies in the physiological functions of SS is widely conducted using clinical tests [10]. Among these tests, Timed Up and Go (TUG), sit-to-stand (STS), and the 10 Meter Walk Test (10MWT) are some of the most prevalent tools employed to evaluate affected motor capabilities [11,12,13]. The TUG test includes a timed sequence of actions, which includes (a) the process of rising from a seated position in a chair, (b) walking 10 feet, turning, and (c) returning to the initial seated position. Similarly, the STS test involves the evaluation of a patient’s ability to transition from a seated position to a standing position and vice versa. In contrast, the 10MWT assesses walking speed and endurance over a designated distance, providing insights into gait patterns and mobility. However, it is noteworthy that conventional applications of tests like TUG, STS, and the 10MWT include measurements of general parameters (such as overall time) and tend to lack the precision required for the evaluation of specific factors, such as a patient’s speed/time for specific body movements and the intricate movement patterns associated with the task. For instance, a TUG time of ~13 s was found to best discriminate between the performance of SS and healthy individuals (HI) [11]. An in-depth examination of the specific motions and patterns exhibited during these tests can offer several insights into the deficiencies caused by stroke. This level of scrutiny not only enhances the detection of affected functions but also holds the potential to refine and optimize targeted rehabilitation strategies.

Cognitive demands, especially those arising during the execution of secondary tasks, have been found to cause irregular body movements. Clinicians have increasingly recognized this aspect, with ~79% of physiotherapists incorporating assessments of cognitive contributions [14]. Researchers have also explored a dual-task (DT) paradigm to account for the intricate interactions between motor and cognitive functions in their evaluations. For instance, Plummer-D’Amato et al. [15] conducted an assessment of the impact of concurrent cognitive tasks, including speech, visuospatial, and working-memory exercises, on gait parameters and observed a decline in the parameters during DT performance. In another study, deterioration in gait parameters was associated with an increased risk of fall [16]. Similarly, a study that evaluated prefrontal cortex activation during DT walking found that the activation may prioritize physical demands in SS but might prioritize cognitive demands in HI [17]. The findings were also aligned with those from a study that suggested the prioritization of balance over cognitive tasks [18]. Overall, studies show potential benefits of incorporating DTs with tests that involve motor activities (like walking/turning) because of their similarity to real-world tasks [19,20,21,22].

Recent strides in sensor technologies have played a pivotal role in enabling detailed performance assessment through motion analysis. Patterson et al. demonstrated the efficacy of motion analysis in assessing post-stroke changes in gait parameters, and Bae et al. showcased the utility of wearable sensors in evaluating conditions like Parkinson’s disease [23,24]. Motion analysis can be helpful in advancing the precision and effectiveness of targeted rehabilitation protocols. Recent studies explored the effects of motor-cognitive DTs [25,26] and their effects during traditional clinical tests such as TUG and the 10MWT [20,27,28,29,30,31,32,33]. Researchers have also implemented motion analysis to detect the effects of stroke on movement patterns during daily activities like walking in the presence of DTs [34,35,36]. Traditionally, movement patterns have been studied using optoelectronic camera-based motion-capture systems, which are laboratory-restrictive despite being highly accurate. To facilitate clinical evaluations, more accessible approaches are being developed. Although video-based pose detection methods can be highly accessible, their capabilities in accurately detecting diverse sets of postures remain a matter of debate. Meanwhile, sensors based on Inertial Measurement Unit (IMU) technology are highly accessible, are sufficiently accurate, and allow field evaluations. Wearable sensors are also present in most modern electronic devices (smartphones, smartwatches, etc.) and can be efficiently used to objectively detect movement variations in clinical populations. By recording movement variations using wearable sensors, it may be possible to detect deficits in specific body functions, enabling more targeted rehabilitation approaches.

Our study is the first to evaluate motor-cognitive DT performance in instrumented clinical tests using multiple wearable motion sensors. The efficiency in detecting affected functions in patients using clinical tests alone, motion analysis alone, or the use of motion analysis in combination with clinical tests remains a point of debate in the stroke community. For instance, one study found that TUG and the step test may allow better prediction of falls than gait speed/static balance measures [37]. The novelty of this study lies in the level of depth in analyzing the impacts of DTs due to the inclusion of wearable sensors. Building on our earlier studies, wherein we employed wearable Inertial Measurement Unit (IMU) sensors to detect movement changes in SS during on-spot turning [38], this study aims to extend our understanding by infusing TUG, STS, and 10MWT clinical tests with detailed motion analysis. This study encompasses multiple objectives: (a) to assess movement patterns between SS and HI during instrumented clinical tests by utilizing multiple wearable sensors; (b) to determine the differences across specific activities within clinical tests and identify the most effective activities to detect motor changes within the common clinical tests; and (c) to assess the impacts of using a DT paradigm on movement patterns in SS. We hypothesize that combining motion analysis (using wearable sensors) with common clinical tests may be more beneficial in identifying specific activities that are more challenging for SS. Furthermore, we expected that inducing additional cognitive demands while performing motor tasks can stress neuro-musculoskeletal systems impacting the individual’s physical performance during tests. The overarching objective of this study is to contribute to the development of effective rehabilitation programs, ultimately enhancing the quality of life and overall well-being of post-stroke patients.

## 2. Materials and Methods

### 2.1. Participants

We recruited 41 participants, including 21 SS/stroke and 20 HI/control. The sample size was determined based on a preliminary power analysis using G*Power software (version 3.1). The results of our analysis demonstrated that for effect size, significance level, and powers of 0.8, 0.05, and 0.8, we needed a sample size of 21 subjects in each group. There were no statistically significant differences in anthropometric measurements (mean (SD)) between the two groups (*p*-values for age, height, weight, and BMI were >0.05), as summarized in Table 1. Prior to the commencement of the study, all participants provided their informed consent by signing a written consent form. This study was conducted in accordance with the approval granted by the Institutional Review Board of both the Rochester Institute of Technology and Rochester Regional Health. Inclusion criteria for the stroke group required participants to be capable of walking independently for distances exceeding 10 m, to have experienced a stroke at least six months prior to their participation, and to be free from any severe medical conditions that might significantly impact their physical performance. Likewise, the healthy control group’s inclusion criteria excluded individuals with neurological diseases or musculoskeletal issues that might affect functional performance during the experiment.

### 2.2. Study Approach 

The body movement of the participants was recorded as they performed clinical tests in two separate conditions, with and without cognitive loading. We selected clinical tests based on the activities involved: sitting/standing, walking, and turning, consisting of TUG, STS, and the 10MWT. All three tests are widely implemented in the stroke research community for evaluating the motor, locomotor, and balance performance of post-stroke patients [7,12,39]. The participants completed five different tests (TUG, STS, the Balance Test with open/closed eyes, and the 10MWT) in a random order. Owing to the scope of this study, we excluded outcomes from the Balance Test. Additionally, for each test, participants either started with a DT or single-task (ST), and these selections were made randomly. To induce cognitive loading during DT, prior studies suggested that subtraction-based tasks rely more on working memory and can impose a higher cognitive load than verbal fluency [40]. Among the methods used to impose cognitive loading, such as reverse counting, reciting alternate alphabets, and naming items (e.g., animals), reverse counting in steps of 3 and 7 is the most common [41,42,43]. However, during pilot studies, our observations showed that post-stroke patients were unable to perform reverse counting in steps of 3 and 7 throughout the tasks because of their challenging nature. Thus, we used a simplified version that involved counting backward from 200 in decrements of 10 while following the prescribed path. If participants reached zero during any tests, they were instructed to restart the counting from 200. Importantly, participants were explicitly guided to ensure that their performance remained independent of the numerical counting task.

#### 2.2.1. Procedural Details of Clinical Assessments

**Figure 1:**
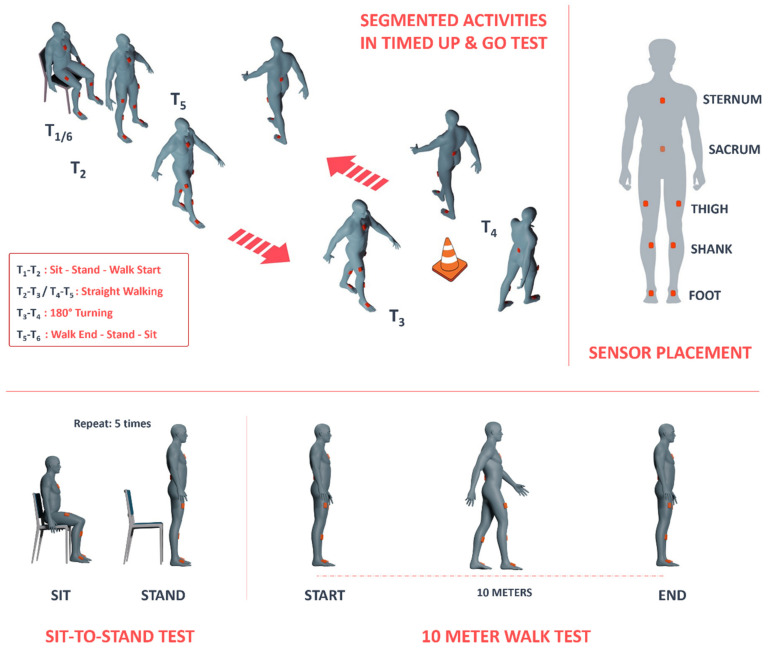
Illustration depicting (top left) segmentation of tasks while performing the Timed Up and Go (TUG) test, (bottom left) the sit-to-stand (STS) test, (bottom right) the 10 Meter Walk Test (10MWT), and (top-right) the placement locations of inertial sensors on the body.

The initial assessment, the TUG test, entailed a sequence of five timed locomotion tasks, which are visually depicted in Figure 1: (1) initiating from a seated position and transitioning to a standing posture; (2) advancing to a designated cone positioned approximately 3 m (equivalent to roughly 10 feet) from the chair’s starting point; (3) executing a turn around the cone; (4) returning to the chair; and (5) performing a turn and reseating on the chair. Each participant completed the TUG test twice, once while concurrently engaging in a cognitive task and once without such DT demands. The selection of the TUG test for this experiment was motivated by its widespread use in clinical settings. Subsequently, the second assessment involved the STS test, during which participants performed five trials of sitting and standing from a chair. Lastly, the third test, the 10MWT, required participants to walk in a straight line for 10 m. Like in the TUG test, participants were asked to count aloud during both the STS and 10MWT assessments.

#### 2.2.2. Experimental Design and Independent Factors

A 2 × 2 experimental design was selected for this study, with independent factors of group (stroke, control) and cognitive load (ST, DT). As participants performed each of the three tests (TUG, STS, 10MWT), their movement was recorded using eight IMUs manufactured by Movella (Xsens, Enschede, The Netherlands) placed on each of the feet, shanks, and thighs and on the sternum and sacrum of the participant in the pre-defined ‘lower-body and trunk’ configuration (Figure 1). Motion data were collected from each sensor simultaneously at a sampling frequency of 100 Hz using the MVN Analyze^®^ software package (Xsens, Enschede, The Netherlands) as the participants performed each of the tests.

### 2.3. Signal Processing and Feature Extraction

We employed a custom-tailored MATLAB (MathWorks, Natick, MA, USA) code to analyze the kinematic data. The data, encompassing segment angles, angular velocity, and linear acceleration, underwent initial preprocessing and extraction using the MVN Analyze^®^ software package (Xsens, Enschede, The Netherlands). To optimize data quality, a 4^th^-order low-pass Butterworth filter with a 5 Hz cut-off frequency was applied to the imported data in MATLAB (version R2022b). For each test, a custom MATLAB code was designed for automatically detecting events in each test, segmentation of the tests for each participant, and feature extraction. Following the code’s execution for each trial, all segmentation processes were confirmed visually by plotting graphs using markers for detected events. In cases where discrepancies arose between the code-generated segmentation and the defined strategy, manual adjustments were made to the segmentation. As the tests encompassed various tasks, each test was subjected to a distinct signal processing, segmentation, and event-detection approach, which will be elaborated on in the subsequent sections.

#### 2.3.1. TUG Test

To conduct a thorough analysis of the TUG test, it was imperative to break the test down into distinct sections. As previously mentioned, we divided the test into five specific sections: (1) standing up from the chair, (2) walking toward the cone, (3) turning around the cone, (4) walking toward the chair, and (5) turning and sitting on the chair. To identify the start and end points of these sections, we defined six key events to be detected in each trial, as illustrated in Figure 1:

T1: Start of the task by the initiation of standing up from the chair

T2: Start of walking toward the cone (also indicating the end of the standing-up phase)

T3: Start of the turn around the cone (also indicating the end of walking toward the cone)

T4: Start of walking toward the chair (also indicating the turning around the cone)

T5: Start of turning and sitting on the chair (also indicating the end of walking toward the chair)

T6: Conclusion of the test by sitting back down in the chair

To identify the T1 and T6 events, we analyzed three signals: thorax resultant angular velocity, right thigh resultant angular velocity, and left thigh resultant angular velocity. The commencement of the task corresponded to the initial movement of any of these body segments. When we observed a significant rise in the signal of any of these segments, we marked that point as T1. Conversely, to determine T6 (the end of the task), we monitored all three signals, expecting them to reach stable, near-zero levels as participants completed the task.

**Figure 2:**
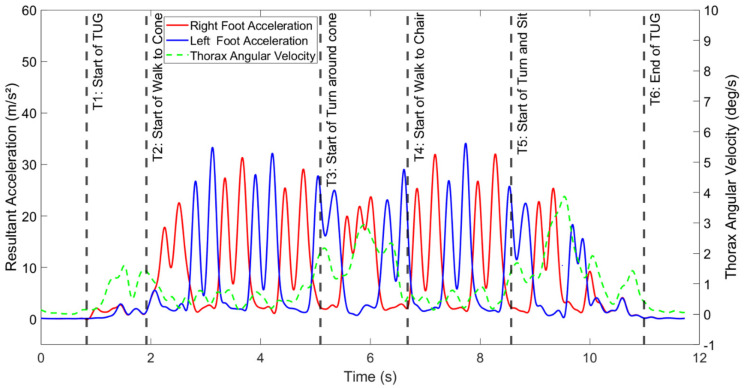
Illustration of the raw signals and segmented sections of the TUG task for a sample participant from the control group.

To detect the T2, T3, T4, and T5 events, we used resultant linear acceleration from both the left and right feet, along with the thorax resultant angular velocity. These events denoted the start and end points of the walking sections (toward the cone and chair), so we aimed to identify them based on the periodic patterns present in the walking signals. The thorax resultant angular velocity signal was used to enhance the accuracy of segmentation during walking during the turning portion, as the commencement and completion of the turn were not clearly discernible from the foot acceleration signals alone. Specifically, we selected the end of a stride (T3) as the point at which a spike in thorax angular velocity occurred, with T4 representing the point when the thorax angular velocity dropped to a minimum after reaching a peak during the turn around the cone. Figure 2 provides a sample of the segmented signals for a participant in the control group, illustrating the effectiveness of this approach. Overall, ten features were calculated, including the total time to complete TUG; the time to perform sit-to-stand, walk toward the cone, turn around the cone, walk toward the chair, and turn and sit; total steps toward the cone and chair; and cadence toward the cone and chair.

#### 2.3.2. STS Test

A sit-to-stand test was implemented to evaluate patients’ ability to transition between sitting and standing positions, and vice versa. As this task primarily involves flexion and extension movements in various body segments, we focused our analysis on the angular velocity of the sensors, rather than linear acceleration. Each test consisted of five repetitions of the sit–stand–sit activity. To segment the signals and measure the duration of each phase, we utilized the resultant angular velocity data from sensors placed on the thighs (upper legs). Within each repetition or cycle of sit–stand–sit, we observed two distinct convex shapes in the angular velocity data. Each of these convex shapes represented either the sitting or standing phases, and we considered the time between two sequences of convex shapes as an event during the segmentation process. For each sit-to-stand trial, we calculated a total of eight features, which included the total time taken for the sit–stand–sit activity, the mean duration of the sit–stand–sit phase, the mean duration of the sit–stand phase, the mean duration of the stand–sit phase, and the root mean square of angular velocity for the thorax, pelvic region, and right/left thighs during the test.

#### 2.3.3. 10MWT

The 10MWT involved a 10 m walk and a comprehensive gait analysis relying on kinematic data. To analyze motion, especially in walking patterns, a crucial step was identifying heel-strike (HS) and toe-off (TO) events. Precise event timing was determined using resultant linear acceleration data collected from sensors on the participants’ shanks, a method consistent with our prior studies [44,45]. The linear acceleration signal displayed two distinct peaks within each stride, one corresponding to TO and the other to HS. These events were identified using segmentation techniques on the sensor data from each shank. It is noteworthy that the gait event could be detected using other sensor data, such as from sensors on the feet or sacrum [46,47]. Subsequently, seven parameters were calculated to quantify various kinematic variables and factors related to walking. These parameters encompassed total walk time, the number of steps during the test, cadence, mean swing total, mean single support portion in strides, gait speed, and stride duration.

### 2.4. Statistical Analysis

To analyze the data, we conducted a repeated-measures analysis of variance (ANOVA). This involved creating models that accounted for the effects of each of the 25 different measures (shown in Table 2) obtained from the tests (TUG: 10 measures, STS: 8 measures, 10MWT: 7 measures). The key independent variables were group (consisting of stroke and control) and cognitive load (encompassing single task (ST) and DT, as well as their interaction). Before performing the analysis, we confirmed that the prerequisites for repeated-measures ANOVA were satisfied, which included verifying that the continuous dependent variable followed a normal distribution (implementing the Shapiro–Wilk method) and ensuring there were no outliers in the repeated measurements. We also calculated effect sizes (partial eta-squared: η^2^) for each analysis, categorizing them as small if η^2^ was less than 0.06, medium if it fell between 0.06 and 0.14, and large if it exceeded 0.14 [48]. In all our analyses, we maintained a significance level of 0.05, and we conducted these statistical computations using the JMP software (version 16.2) from the SAS Institute in North Carolina, USA.

## 3. Results

The statistical analysis presented in Table 2 revealed significant main effects for both group and cognitive load on various key outcome measures in the TUG, STS, and 10MWT assessments. Although parameters such as STS time and RMS angular velocity were primarily influenced by group but not by cognitive load, others, like cadence and swing time, were significantly affected by cognitive load but not by group membership. Findings show an absence of significant interactions between group and cognitive load. Effect sizes for group-related main effects were substantial, particularly in multiple TUG parameters, including total time, walking duration, and turning sections. In contrast, the main effects of cognitive load generally had minor to moderate effects on the measured outcomes. The introduction of a cognitive DT further exacerbated participants’ physical limitations during the clinical assessment tests.

Table 3 displays the mean (SD) values of key outcome measures for both single-task (ST) and dual-task (DT) conditions within each group. The analysis highlighted specific activities that posed significantly greater challenges for stroke survivors. In particular, they exhibited a 23% increase in the total time needed to complete the TUG test, with notable variations in the TUG subsections: 20% longer for walking to the cone, 15% for turning around the cone, and 29% for turning and sitting back down, with the most substantial difference observed in the turn and sit subsection. In the STS test, stroke survivors exhibited a 20% reduction in trunk angular velocity, and in the 10MWT, they recorded a 21% longer walk time and a reduced gait speed when compared with the control group.

**Figure 3:**
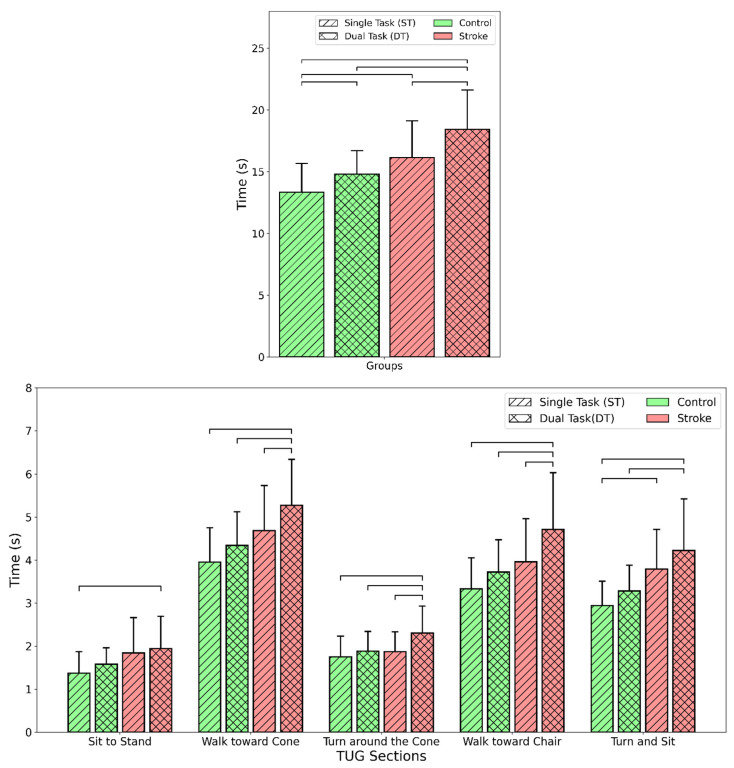
Comparison of the time taken to complete the tasks for different groups (stroke and control) and cognitive loads (single task and dual task). (Top) TUG total time and (bottom) times for the five subsections of TUG. The bars connected to the lines are significantly different (*p*-value < 0.05). Note: Each set of four bars (from left to right) in each section shows the results for the control group under ST and DT conditions and the stroke group under ST and DT conditions, respectively.

**Figure 4:**
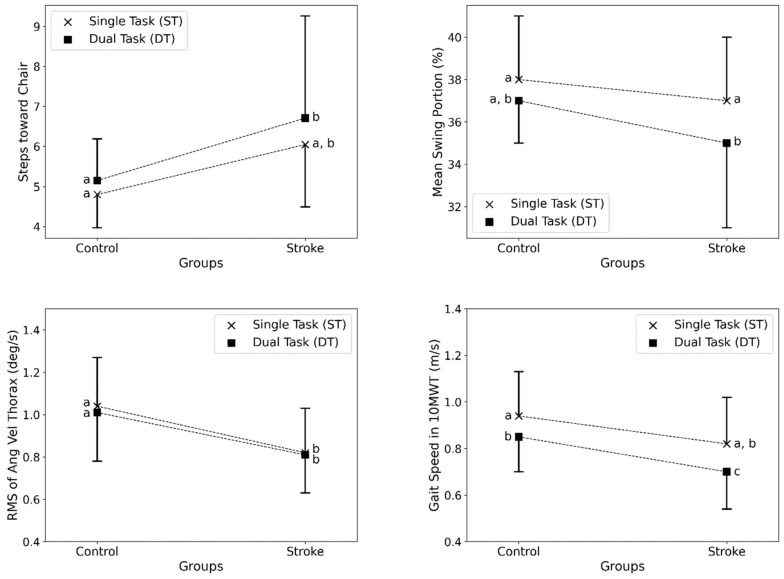
Comparison of key outcome measures between stroke survivors and healthy individuals in single-task (ST) and dual-task (DT) conditions. (Top left) Steps toward the chair in the TUG test, (top right) mean swing portion of strides in the 10MWT (%), (bottom left) RMS of thorax angular velocity in STS (deg/s), and (bottom right) gait speed in the 10MWT (m/s). Error bars indicate standard deviation. The points with no common letter labels (i.e., a, b, and c) were significantly different (*p*-value < 0.05) in the post-hoc analysis (lack of common letters between the control and stroke groups denotes statistical significance). The dotted lines connect the outcomes of the relevant cognitive loads in control and stroke groups.

The results are further illustrated in Figure 3 and Figure 4, which compare key outcome measures between stroke survivors and healthy individuals in ST and DT conditions. Figure 3 shows that stroke survivors took significantly longer to complete the total TUG test and its subsections, especially for turning activities, compared with healthy individuals. DTs increased times across both groups. Figure 4 shows that stroke survivors had more steps toward the chair in the TUG test, reduced swing portion in the 10MWT, lower trunk velocity during sit-to-stand transitions, and slower gait speed in the 10MWT compared with controls. Implementing DTs worsened these metrics for both groups. The figures demonstrate deficits in stroke survivors across sitting, standing, walking, and turning movements, which were further impacted by cognitive demands during the clinical assessment tests.

## 4. Discussion

In this study, we examined the movement deficits in post-stroke patients by instrumenting the common clinical TUG, STS, and 10MWT assessments with the help of wearable sensors. The novelty of our approach was: (i) to enable detailed assessment of body movement as participants performed the tasks within these tests, and (ii) to assess the impact of cognitive loading while performing the tests by incorporating single and motor-cognitive DT conditions. This is the first study to conduct in-depth motion analysis to assess the impacts of DTs in common clinical tests by implementing multiple wearable motion sensors. The outcomes of the study revealed significant impairments in SS compared with HI as they performed tests that included a combination of ADLs, particularly sit/stand, walking, and turning. Notably, our findings (Table 3) also showed that the addition of cognitive loading in the DT conditions substantially impacted body movements in SS, highlighting the potential impact of divided attention on physical performance. Prior studies demonstrated that inducing additional cognitive loading can lead to decreased performance in completing tasks [15,18,20,34]. The findings of this study are consistent with earlier findings, and the introduction of a secondary cognitive task during the tests further worsened the motor impairments in SS.

Substantially greater variability was seen in the SS compared with the HI for several of the extracted kinematic features across the clinical tests (see Table 3). SS took 23% longer to complete the TUG test, reflecting significant challenges in the performance of sequential motor tasks. Segmentation of the TUG test into walking, turning, and sit/stand was beneficial for identifying differences between SS and HI, especially for assessing the impacts of cognitive loading [29]. As depicted in Figure 3, detailed segmentation revealed specific sections that were more demanding, such as the 29% increase in the time taken for the final turn and sit subsection of the TUG test. The TUG test best detected mobility limitations in stroke survivors, with total time increased by 23% compared with controls. Further analysis of TUG subsections provided insights into which activities were specifically challenging (Figure 3). Turning ability in SS was objectively evaluated by Hollands et al., 2014, wherein the mean time to turn during TUG was higher in the SS than in the HI (2.12 vs. 1.99 s). This study yielded similar outcomes, with the turn around the cone section being 15% longer in SS compared with HI, indicating turning deficiencies. The final turn and sit subsection had an even greater 29% increase, suggesting transitional movements like sitting are especially demanding. SS also took 20% longer for the straight walk sections. Detailed TUG analysis using wearable sensors, as conducted in this study, demonstrates the value of segmenting clinical tests to reveal specific problematic activities compared with using total time alone.

Consistent with the findings of our earlier study [49], cognitive loading through DT in this study impacted TUG performance in both groups, but more so in SS. DTs increased total TUG time by 20% in SS versus 11% in HI. In a previous study, cognitive loading in TUG increased the duration of the test by ~3.7–5.9 s (total time ~17–21 secs with cognitive loading), similar to the findings (Figure 3) reported in our study [20]. Examining the subsections showed that turn time was disproportionately affected, increasing by 31% under DT conditions in stroke survivors. Turning requires the integration of multiple sensorimotor processes, including postural transitions, asymmetric limb coordination, and spatial navigation [35,38]. DTs may overwhelm these processes in SS in the regions of the brain that govern cognition, attention, and motor control. Targeted training focusing on turning and transitions under cognitive load may improve TUG performance. Overall, instrumented analysis of TUG with DTs allowed robust assessment of the motor-cognitive capabilities affected by stroke.

During the STS test, the total test time under DT conditions had a standard deviation of 6.83 s in SS vs. 4.25 s in HI. This highlights the considerable heterogeneity in functional disabilities and compensatory movement strategies adopted by the stroke population. This result could be due to several factors, such as the location or extent of the injury (brain lesion), severity of the stroke, and duration since stroke occurrence [16]. The obtained kinematic features that showed pronounced deficits and variability in SS vs. HI show potential as sensitive metrics for tracking longitudinal recovery and improvements during rehabilitation. For example, the STS trunk angular velocity detected mobility limitations related to transitional movements [20,34]. Clinically, these objective instrumented measures could inform individualized prognoses and therapies tailored to each patient’s specific functional challenges revealed during standardized tests. Furthermore, the pronounced deficits in trunk angular velocity during sit-to-stand in stroke survivors point to weakened core muscles and postural instability after stroke. STS transitions require dynamic balance capabilities, which may be further compromised by divided attention demands [37,50]. It is also possible that the greater attentional cost for maintaining stability could manifest in the form of affected kinematic parameters, such as reduced trunk velocity.

The absence of significant main effects of group or cognitive load on metrics like total time and phase durations during the STS test may be attributed to several factors. High inter-individual variability in transitional movements between participants likely contributed to considerable variability in STS performance that may have masked group differences. The straightforward sit-to-stand motion may have posed less of a motor challenge compared with more dynamic tasks like turning and walking for this cohort of stroke survivors. Furthermore, multiple repetitions of the STS task could have allowed participants to optimize their performance through practice effects, and the counting DT may not have sufficiently challenged cognitive-motor integration during the primarily motor sit-stand activity. Importantly, the limited sample size of stroke survivors may have reduced the statistical power to detect group or cognitive load effects. These factors potentially explain the lack of significant differences and indicate that STS test parameters may not differentiate stroke survivors from controls as well as other mobility metrics in certain populations and testing conditions.

The 10MWT revealed considerable variability in spatiotemporal gait metrics, including cadence, stride length, stance/swing ratios, and gait speed in stroke survivors. Similar outcomes were reported in [15], wherein cognitive loading affected gait speed, stride time, average stride length, and cadence during the 10MWT test. This could mean that stroke can disturb locomotor rhythmicity and symmetry owing to affected brain functions that map to leg motor functions, as seen in the form of alterations in gait parameters [27,51,52]. SS exhibited impaired gait speed, reduced cadence, shortened swing phase, and longer double support time compared with HI, as depicted in Table 3. DT likely shifts priority toward maintaining safe walking patterns, resulting in conservative alterations in cadence, swing time, and support phases in SS [52,53]. The changes in gait metrics may signify the recruitment of cognitive resources for conscious control and monitoring of movements. DT costs were higher across spatiotemporal gait parameters in stroke survivors, reiterating their limited cognitive reserve.

Among the studied clinical tests, 10MWT provided unique insights into spatiotemporal gait impairments in SS, such as reduced gait speed, cadence, stride length, and swing ratio. These gait metrics capture characteristics that may not be evident from total time alone during short walks in the TUG test. However, the 10MWT lacks the multi-tasking components involved in activities of daily living. Therefore, integrating the 10MWT straight path walking into an instrumented TUG test could offer advantages for both measures. According to the results of this study, we propose a modified TUG starting with the patient already standing, then walking 10 m, turning 180 degrees around a cone, walking 10 m back, and ending by sitting down. This would allow detailed quantification of gait parameters over an extended distance along with an assessment of turning under cognitive load. The sit-to-stand transition may provide less added value based on our findings. Eliminating this portion would shorten test time and reduce the complexity of the analysis while still capturing the key markers of mobility deficits. Thus, a modified instrumented TUG incorporating gait analysis over 10 m and a DT turn component may optimally assess motor-cognitive capabilities affected by stroke. Further research is needed to develop and validate such an integrated clinical assessment.

The instrumented metrics obtained in this study hold potential for translation to clinical practice to inform individualized rehabilitation. For instance, the disproportionate DT deficits in turn time could indicate the need for targeted training focused on transitional movements and navigation under divided attention in stroke survivors [54]. Likewise, reduced trunk velocity during repeated sit–stand transitions points to weakened core muscles requiring specific strengthening. Quantifying such functional limitations through sensor-based assessment during standardized tests allows customized therapies tailored to patient weaknesses. The metrics showing pronounced stroke-related impairments like TUG turn time and 10MWT gait speed could also serve as sensitive outcomes to track subtle longitudinal improvements resulting from interventions. Collaborations spanning engineers, clinicians, and data scientists would allow the development of predictive models leveraging these metrics to forecast fall risk or functional prognosis [55,56]. Overall, the methodology presented contributes to future technologically enabled precision rehabilitation paradigms and could optimize the quality of care. Integration of quantified instrumented clinical tests into immersive biofeedback platforms might actively engage patients in self-rehabilitation. Further research should explore such clinical translation while addressing practical challenges involving patient compliance, clinician adoption, and ethical considerations around data privacy.

This study, while demonstrating a detailed evaluation of motor-cognitive capabilities impacted by stroke through the integration of standardized clinical tests, motion analysis, and DT paradigms, is not without its constraints. The relatively small sample size, though a common limitation in studies involving clinical populations, may affect the generalizability of the findings. In addition, there was a marginally significant difference between the ages of the two groups that should be addressed in future studies to allow more reliable results. Furthermore, the potential benefits of incorporating additional sensor modalities such as electromyography to gain insights into neuromuscular control warrant consideration. To further enhance our understanding, future research endeavors should explore longitudinal studies that follow patients through rehabilitation, offering a dynamic perspective on how the observed metrics evolve over time and their therapeutic implications. These endeavors have the potential to refine rehabilitation strategies and, in turn, contribute to an improved quality of life for SS.

## 5. Conclusions

In this study, we used wearable sensors to perform a detailed study of movement alterations in stroke survivors during common clinical tests (TUG, STS, and the 10MWT). Using motion analysis, we effectively segmented the tests into specific activities and identified critical deficits in SS, such as reduced trunk velocity in STS, altered gait patterns in TUG, and asymmetric strides in the 10MWT. The outcomes of our study show that SS took significantly longer to complete the clinical tests, especially the TUG total time and turning sections, compared with HI. When DT conditions were imposed, impairments in SS were exacerbated across all tests, indicating the impact of divided attention on physical performance. This showed that the combination of instrumented clinical tests and DT paradigms allows robust assessment of motor-cognitive capabilities affected by stroke and provides insights into functional disabilities post-stroke. Overall, detailed quantification of specific task segments and DT conditions can inform targeted rehabilitation strategies. Future work should explore predictive modeling of fall risks and prognoses based on these instrumented metrics. The proposed methods can be translated to clinical practice for the objective evaluation of patient status and recovery. Overall, this research lays the groundwork for technologically enabled, precision rehabilitation to improve outcomes in stroke survivors.

## Figures and Tables

**Table 1:** Demographic data for study participants.

Parameters	Stroke (N = 21)	Control (N = 20)	*p*-Value (*t*-Test)
Gender	11 males 10 females	8 males 12 females	-
Age (year)	66 (10)	60 (8)	0.053
Height (cm)	173.8 (8.4)	172.6 (9.6)	0.669
Weight (kg)	86.3 (14.7)	81.8 (18.2)	0.39
BMI (kg/s^2^)	28.5 (4)	27.2 (3.9)	0.288

**Table 2:** Main and interaction effects of the group (stroke and control) and cognitive load (single-task (ST) and dual-task (DT)) on the measures from TUG: Timed Up and Go, STS: sit-to-stand, and 10MWT: 10 Meter Walk Test. (Note: The three columns headed Group, CL, and Group × CL show the *p*-values for the null hypotheses of no significant effect of these factors on the dependent factors. The partial effect size (η^2^) of each relevant effect analysis is reported in the column to its right. CL: cognitive load).

Measures	Group	η^2^	CL	η^2^	Group × CL	η^2^
**TUG Test**						
Total time (s)	**<0.001**	0.244 (L)	**<0.001**	0.085 (M)	0.124	0.004 (S)
Sit-to-stand (s)	**0.036**	0.079 (M)	0.056	0.023 (S)	0.542	0.002 (S)
Walk toward cone (s)	**0.003**	0.166 (L)	**<0.001**	0.059 (S)	0.396	0.003 (S)
Turn around the cone (s)	**0.035**	0.069 (M)	**0.005**	0.068 (M)	0.116	0.020 (S)
Walk toward chair (s)	**0.006**	0.152 (L)	**<0.001**	0.067 (M)	0.140	0.007 (S)
Turn and sit (s)	**0.001**	0.206 (L)	**0.008**	0.033 (S)	0.835	<0.001 (S)
Steps toward cone	**0.002**	0.202 (L)	0.113	0.005 (S)	0.371	0.001 (S)
Steps toward chair	**0.005**	0.170 (L)	**0.022**	0.018 (S)	0.440	0.001 (S)
Cadence toward cone (step/min)	0.916	0.001 (S)	**<0.001**	0.107 (L)	0.592	<0.001 (S)
Cadence toward chair (step/min)	0.434	0.014 (S)	**0.002**	0.058 (S)	0.405	0.005 (S)
**STS (×5) Test**						
Total time (s)	0.289	0.019 (S)	0.150	0.002 (S)	0.215	0.008 (S)
Mean STS (s)	0.289	0.019 (S)	0.150	0.002 (S)	0.215	0.008 (S)
Mean STS up (s)	0.166	0.038 (S)	0.353	0.001 (S)	0.288	0.005 (S)
Mean STS down (s)	0.481	0.006 (S)	0.061	0.002 (S)	0.199	0.009 (S)
RMS of ang vel thorax (deg/s)	**0.003**	0.187 (L)	0.390	<0.001 (S)	0.464	0.001 (S)
RMS of ang vel pelvis (deg/s)	0.182	0.037 (S)	0.285	0.001 (S)	0.143	0.005 (S)
RMS of ang vel right thigh (deg/s)	0.569	0.004 (S)	**0.026**	0.004 (S)	0.204	0.005 (S)
RMS of ang vel left thigh (deg/s)	0.391	0.013 (S)	**0.032**	0.004 (S)	0.261	0.005 (S)
**10MWT**						
Walk time (s)	**0.010**	0.135 (M)	**<0.001**	0.064 (M)	0.257	0.005 (S)
Steps	**0.019**	0.124 (M)	**<0.001**	0.029 (S)	0.504	<0.001 (S)
Cadence (step/min)	0.184	0.036 (S)	**<0.001**	0.070 (M)	0.152	0.007 (S)
Mean swing total (%)	0.133	0.050 (S)	**<0.001**	0.037 (S)	0.076	0.007 (S)
Single support (%)	0.781	0.001 (S)	**0.044**	0.025 (S)	0.178	0.008 (S)
Gait speed (m/s)	**0.012**	0.124 (M)	**<0.001**	0.079 (M)	0.528	0.001 (S)
Stride duration (s)	0.111	0.050 (S)	**<0.001**	0.067 (M)	0.113	0.010 (S)

**Table 3:** Mean (SD) values of the key outcome measures of the three tests (TUG: Timed Up and Go, STS: sit-to-stand, and 10MWT: 10 Meter Walk Test) for each group in single-task (ST) and dual-task (DT) conditions. Statistically significant values are denoted by: *. Ranges are defined as follows: *p*-value > 0.05: -, *p*-value ≤ 0.05: *, *p*-value ≤ 0.01: **, *p*-value ≤ 0.001: ***).

Measures	Control (N = 20)		Stroke (N = 21)		Group *(Control ST* vs. *Stroke ST*)	CL *(Control DT* vs. *Stroke DT*)
	Control ST	Control DT	Stroke ST	Stroke DT		
TUG Test						
Time total (s)	13.34 (2.34)	14.8 (1.91)	16.15 (2.97)	18.44 (3.17)	***	***
Sit-to-stand (s)	1.37 (0.5)	1.58 (0.38)	1.84 (0.82)	1.94 (0.75)	*	-
Walk toward cone (s)	3.95 (0.8)	4.34 (0.78)	4.68 (1.05)	5.27 (1.07)	**	***
Turn around the cone (s)	1.75 (0.48)	1.88 (0.46)	1.87 (0.46)	2.3 (0.63)	*	**
Walk toward chair (s)	3.33 (0.72)	3.72 (0.75)	3.96 (1)	4.71 (1.32)	**	***
Turn and sit (s)	2.94 (0.57)	3.28 (0.6)	3.79 (0.92)	4.22 (1.2)	**	**
Steps toward cone	6.05 (1)	6.15 (1.04)	7.19 (1.54)	7.57 (1.54)	**	-
Steps toward chair	4.8 (0.83)	5.15 (1.04)	6.05 (1.56)	6.71 (2.55)	**	*
Cadence toward cone (step/min)	93.21 (12.11)	85.43 (7.61)	92.78 (9.97)	87.47 (14.44)	-	***
Cadence toward chair (step/min)	88.08 (12.77)	83.69 (10.61)	92.21 (11.17)	85.19 (15.83)	-	**
**STS (×5) Test**						
Time total (s)	17.81 (3.23)	19.06 (4.25)	20.04 (6.59)	20.6 (6.83)	-	-
Mean STS (s)	3.56 (0.65)	3.81 (0.85)	4.01 (1.32)	4.12 (1.37)	-	-
Mean STS up (s)	1.75 (0.32)	1.85 (0.42)	2.01 (0.65)	2.06 (0.63)	-	-
Mean STS down (s)	1.81 (0.35)	1.96 (0.45)	2 (0.75)	2.06 (0.79)	-	-
RMS of ang vel thorax (deg/s)	1.04 (0.23)	1.01 (0.23)	0.82 (0.21)	0.81 (0.18)	**	-
RMS of ang vel pelvis (deg/s)	0.91 (0.26)	0.86 (0.23)	0.78 (0.28)	0.78 (0.25)	-	-
RMS of ang vel right thigh (deg/s)	1.03 (0.21)	0.97 (0.21)	0.97 (0.24)	0.95 (0.21)	-	*
RMS of ang vel left thigh (deg/s)	1.03 (0.21)	0.98 (0.21)	0.96 (0.23)	0.93 (0.2)	-	*
**10MWT**						
Walk time (s)	10.98 (2.12)	12.18 (2.36)	12.94 (3.41)	15.1 (3.81)	**	***
Steps	17.7 (2.3)	18.8 (2.63)	20.14 (4.04)	21.57 (4.42)	*	***
Cadence (step/min)	98.05 (8.87)	93.83 (9.1)	95.58 (13.23)	87.5 (12.81)	-	***
Mean swing total (%)	0.38 (0.03)	0.37 (0.02)	0.37 (0.03)	0.35 (0.04)	-	***
Single support (%)	0.6 (0.06)	0.6 (0.04)	0.61 (0.04)	0.58 (0.06)	-	*
Gait speed (m/s)	0.94 (0.19)	0.85 (0.15)	0.82 (0.2)	0.7 (0.16)	*	***
Stride duration (s)	1.21 (0.12)	1.27 (0.13)	1.26 (0.19)	1.38 (0.21)	-	***

## Data Availability

The raw data supporting the conclusions of this article will be made available by the corresponding authors upon reasonable request.
